# Gut microbiota signatures differentiate trajectory-defined response phenotypes and predict self-management outcomes in irritable bowel syndrome

**DOI:** 10.3389/frmbi.2026.1884540

**Published:** 2026-07-29

**Authors:** Jie Chen, Aolan Li, Weizi Wu, Wanli Xu, Tingting Zhao, Angela R. Starkweather, Leonel Rodriguez, Ming-Hui Chen, Xiaomei S. Cong

**Affiliations:** 1 College of Nursing, Florida State University, Tallahassee, FL, United States; 2 Yale School of Nursing, Orange, CT, United States; 3 Department of Statistics, University of Connecticut, Storrs, CT, United States; 4 School of Nursing, University of Connecticut, Storrs, CT, United States; 5 School of Nursing, Columbia University, New York, NY, United States; 6 Division of Nursing Science, Rutgers School of Nursing, New Brunswick, NJ, United States; 7 Yale School of Medicine, New Haven, CT, United States

**Keywords:** chronic pain, gut microbiota, irritable bowel syndrome, machine learning, precision health

## Abstract

**Introduction:**

Heterogeneity in symptom presentation and treatment response in irritable bowel syndrome (IBS) remains poorly understood. This analysis from a randomized controlled trial (NCT03332537) aims to identify symptom-trajectory phenotypes and determine whether gut microbiota composition and function distinguish these phenotypes and predict multidimensional responses to IBS pain self-management interventions.

**Methods:**

Participants with longitudinal data (n = 62) were analyzed using longitudinal k-means clustering based on trajectories of measures in IBS quality of life (QOL), Brief Pain Inventory (BPI), and neuropsychological outcomes (anxiety, applied cognition, depression, fatigue, global health, positive affect, and sleep disturbance) over 12 weeks. Bayesian Additive Regression Trees (BART) models were used to identify baseline microbial taxa and pathways predictive of longitudinal changes in QOL, BPI pain interference, and severity.

**Results:**

Two distinct trajectory-defined response phenotypes were identified: a Constrained Response Phenotype (Phenotype A, n = 35) and an Adaptive Multidomain Response Phenotype (Phenotype B, n = 27). At baseline, Phenotype B showed lower pain severity and interference, but higher levels of anxiety, depression, and fatigue compared to Phenotype A. Over 12 weeks, both phenotypes showed improvements in pain outcomes (all p < 0.05), but only Phenotype B demonstrated broad improvements across neuropsychological domains and QOL (all p < 0.05). Phenotype A exhibited more limited improvements and worsening in several neuropsychological domains. Nominal differences in predicted functional pathways were observed, including pathways related to xenobiotic degradation, amino acid metabolism, bile secretion, and immune-related processes (all raw p < 0.05). Although predicted functional pathway differences were not significant after correction for multiple testing, phenotype-specific microbial taxa and functional features were identified as predictors of treatment response in BART models. In Phenotype A, genera such as *Alistipes* and *Sutterella* were consistently identified across models, whereas in Phenotype B, predictors included *Phascolarctobacterium*, *Collinsella*, and *Parabacteroides*.

**Conclusions:**

IBS patients exhibit distinct multidimensional response patterns associated with distinct clinical and microbiome profiles. Baseline gut microbial characteristics may serve as potential biomarkers of heterogeneous treatment response in young adults with IBS, supporting a microbiome-based approach to categorize patients and improve personalized self-management strategies in IBS.

## 1 Introduction

Irritable bowel syndrome (IBS) is a prevalent disorder of gut–brain interaction affecting up to 20% of adults worldwide (Lovell and Ford, 2012a; Oka et al., 2020; Palsson et al., 2020; Arif et al., 2025; Ballena-Caicedo et al., 2025), typically manifests in early adulthood and affects more women than men (Lovell and Ford, 2012a; Lovell and Ford, 2012b; Black et al., 2020b; Oka et al., 2020; Palsson et al., 2020; Arif et al., 2025; Ballena-Caicedo et al., 2025). It is a leading cause of healthcare utilization and work absenteeism, accounting for more than $21 billion annually in direct and indirect costs in the United States alone (Leong et al., 2003; Longstreth et al., 2003; Black and Ford, 2020; Bosman et al., 2023; Peery et al., 2025). Despite its high prevalence and economic burden, effective long-term management remains limited (Khan and Chang, 2010; Camilleri, 2021; Lacy et al., 2021). Pharmacologic therapies yield modest and inconsistent benefits and often fail to address the multidimensional nature of IBS, including co-occurring pain, psychological distress, and impaired quality of life (QOL) (Drossman, 2016; Ford et al., 2017; Black et al., 2020a; Piovezani Ramos and Camilleri, 2023; Staudacher et al., 2023). Patients with IBS frequently report prolonged difficulty identifying effective self-management strategies, contributing to sustained pain and reduced QOL (Amouretti et al., 2006; Mönnikes, 2011; Cong et al., 2018a; Cassar et al., 2020). These challenges are further compounded by substantial inter-individual variability in symptom presentation and treatment response, highlighting the need to identify distinct response patterns that may inform more precise, mechanism-based, non-pharmacologic approaches within a whole-person health framework.

The gut microbiome represents a central pathway linking self-management behaviors to symptom regulation in IBS (45,75–77). Beyond taxonomic composition, microbial functional capacity, such as pathways involved in short-chain fatty acid production, aromatic amino acid metabolism, bile acid transformation, and neuroactive compound synthesis, plays a critical role in modulating host neuroimmune and neuroendocrine processes (78–81). These microbial metabolites influence vagal signaling, inflammation, intestinal permeability, and gastrointestinal motility, all of which are implicated in IBS pathophysiology. In parallel, stress-related alterations in autonomic and immune function can reshape the gut environment and microbial activity, suggesting a bidirectional regulatory system. Self-management interventions, particularly those targeting stress and behavioral regulation, may act through this gut–brain axis by inducing downstream shifts in microbial function. However, whether variability in microbial composition and functional capacity contributes to differences in longitudinal symptom trajectories and intervention responsiveness remains poorly understood.

A critical gap remains in understanding how heterogeneity in multidimensional symptom trajectories relates to underlying biological mechanisms, particularly microbial functional dynamics. IBS is characterized by substantial inter-individual variability across pain, psychological symptoms, and quality of life, yet most prior studies rely on average treatment effects and do not capture distinct patterns of response over time. Person-centered approaches, such as trajectory-based clustering, offer an opportunity to identify subgroups of individuals with distinct longitudinal response profiles that integrate these domains. Identification of such phenotypes may provide a more clinically meaningful representation of intervention response and uncover latent regulatory differences not apparent in group-level analyses. Moreover, the extent to which gut microbiota composition and function distinguish these trajectory-defined phenotypes, and whether such differences reflect underlying regulatory mechanisms rather than treatment assignment, remains unclear.

To address these gaps, this study leveraged longitudinal data from a randomized controlled trial to: (1) identify multidimensional symptom trajectory phenotypes using repeated measures of QOL, pain, and neuropsychological outcomes; (2) determine whether gut microbiota composition and inferred functional pathways differ between these phenotypes; and (3) evaluate whether baseline microbial features predict intervention response using a machine learning framework. By integrating longitudinal phenotyping, microbiome profiling, and predictive modeling, this study aims to advance microbiome-informed stratification approaches for personalized self-management in IBS and to characterize response heterogeneity beyond treatment-group effects.

## 2 Methods

### 2.1 Study design and participants

Details of the design, rationale, and primary results of the RCT (NCT03332537) have been published (Cong et al., 2018b; Chen et al., 2022). The RCT protocol was approved by the University Institute Review Board (IRB). Informed consent was acquired from each participant. Privacy was maintained during the entire data collection and management process. Young adults with IBS (N = 80) were recruited in the northeastern United States for this RCT. Young adults were enrolled in the study if they were (1)18–29 years of age; (2) able to read and speak in English; (3) able to access the internet; (4) having a clinical diagnosis of IBS according to the Rome III or IV criteria from a healthcare provider (for IBS subjects only). Subjects were excluded if they had (1) chronic pain conditions (e.g., chronic pelvic pain, headache, back pain, etc.); (2) severe mental health conditions; (3) celiac disease or inflammatory bowel disease; (4) infectious diseases; (5) diabetes mellitus; (6) injury or open skin lesions on the non-dominant arm; (7) history of substance abuse or regular opioid use; (8) history of prebiotics/probiotics or antibiotics use in the past months (Chen et al., 2022). Women during pregnancy or within 3 months postpartum period were also excluded. Eligible participants with IBS were allocated to the intervention groups (Online Modules Plus vs. Online Modules) using a stratified, blocked randomization scheme. The full description of randomization and blinding was previously described in the published study protocol (Cong et al., 2018b). De-identified data were used in this analysis.

### 2.2 Assessment of variables

This analysis leveraged data collected in the RCT (NCT03332537) (Cong et al., 2018b; Chen et al., 2022). Measurements in the RCT (NCT03332537) included demographic characteristics, pain, quality of life, and neuropsychological symptoms. The demographic characteristics include age, sex, ethnicity, race, education level, and other factors measured using the National Institute of Nursing Research common data elements (Page et al., 2018). Questionnaires were completed online through the REDCap system using a laptop or iPad. Participants with IBS completed all questionnaires at enrollment (W0, 0 weeks) and at 6- and 12-week follow-up visits (W6 and W12, respectively).

#### 2.2.1 Pain

Pain severity and interference were measured using the Brief Pain Inventory (BPI). The BPI uses 0–10 rating scales, with higher scores indicating more severe pain (Keller et al., 2004).

#### 2.2.2 Quality of life

IBS quality of life was measured by a 34-item IBS-specific quality of life instrument (Hahn et al., 1997). This instrument was designed to capture patients’ perceptions of their daily functions interfered with by IBS. There are 8 subscales in this five-point Likert instrument. The score range for this scale is 0-100, with higher scores indicating better quality of life.

#### 2.2.3 IBS-related neuropsychological measures

Neuropsychological outcomes were defined as patient-reported symptoms and psychosocial health indicators that capture the interaction of psychological, neurological, and behavioral functioning (Schroeder et al., 2019). IBS-related neuropsychological outcomes, including anxiety, applied cognition, depression, fatigue, global health, positive affect, and sleep disturbance, were measured using the NIH Patient-Reported Outcomes Measurement Information System (PROMIS^®^) and scored according to the system instructions (Cella et al., 2010). A higher T score on the PROMIS measurement indicated a higher intensity of the measured concept, and a mean score greater than 55 indicates that a study subject experienced a significantly higher intensity of the symptom/well-being than the healthy reference population, according to the PROMIS guide.

#### 2.2.4 Stool sample collection and gut microbiota sequencing

Participants collected stool samples at home following our previous protocol using the OMNIgene^®^-gut (DNA Genotek, Ottawa, Canada) kit (Barandouzi et al., 2022; Chen et al., 2023b). Training on collecting the samples at home was provided to the participants by research personnel. After collection, participants were instructed to mail the samples back to the research laboratory within a week of the baseline visit, using a pre-labeled, pre-paid envelope with a tracking barcode. Once received by the laboratory, the stool samples were aliquoted into small tubes and stored at -80 °C in a freezer for bulk processing. A total of 0.25 g of stool from each participant was placed into a bead tube and sent out for DNA extraction and sequencing. The 16S rRNA V4 region amplicon was sequenced by the Illumina MiSeq 2000 platform (Illumina, San Diego, CA) at the University of Connecticut (UConn) Microbial Analysis, Resource, and Services laboratory following our previous protocol (Chen et al., 2023b). The raw 16S rRNA sequencing data were processed by the Mothur 1.43.0 software following the analysis pipeline of Miseq (http://www.mothur.org/wiki/MiSeq_SOP) to obtain the taxonomy and diversity of the gut microbiota. Paired-end sequences were combined into contigs and aligned against the SILVA 132 V4 16S rRNA gene reference alignment database, with poor-quality sequences removed. Operational taxonomic units (OTUs) were identified at a 97% identity (Chen et al., 2021; Chen et al., 2023a).

Microbial functional profiles were inferred from 16S rRNA gene sequences using Tax4Fun2, which predicts gene family abundances based on reference genomes (Wemheuer et al., 2020). Predicted functions were annotated as Kyoto Encyclopedia of Genes and Genomes orthologs (KOs) and aggregated for downstream pathway-level analyses (Kanehisa et al., 2023).

### 2.3 Data analysis

All statistical analyses were conducted using R version 4.5.1. Longitudinal k-means clustering (KML) (Genolini and Falissard, 2010; Genolini et al., 2015) was applied to the IBS quality of life (QOL), Brief Pain Inventory (BPI), and neuropsychological outcomes (anxiety, applied cognition, depression, fatigue, global health, positive affect, and sleep disturbance) measured by the Patient-Reported Outcomes Measurement Information System (PROMIS) over 12 weeks. Candidate cluster solutions were evaluated using the Calinski-Harabasz criterion (Caliński and Harabasz, 1974). The retained two-cluster solution classified participants into Phenotype A (n = 35) and Phenotype B (n = 27). To assess clustering stability, we conducted a repeated KML sensitivity analysis across 1,000 reruns, with 20 redrawings per rerun, evaluating candidate k values from 2 to 6. The Calinski-Harabasz criterion selected k = 2 in all 1,000 reruns (100.0%). The group-size distribution varied across random starts; however, the submitted 35/27 split was the most frequent, occurring in 872 of 1,000 reruns (87.2%).

Exploratory analyses in longitudinal gut microbiota features, including the diversity indices, abundance of genus, and predicted pathways among participants with available stool samples at weeks 0, 6, and 12, were examined using limma (Ritchie et al., 2015). The interaction term (cluster × visit) of gut microbiota features was also tested by limma models (Ritchie et al., 2015). Exploratory genus-level differential abundance analyses using ANCOM-BC2 (Lin and Peddada, 2024). Pathways highlighted by phenotype and visit interaction were visualized in a heatmap.

Bayesian Additive Regression Trees (BART) (Sparapani et al., 2021) were employed to investigate the relationship between predictors and the outcomes of QOL, BPI pain interference, and pain severity. A forward prediction model estimates the change in outcomes from baseline to a future visit using gut microbiota predictors from baseline visit (e.g., baseline predictors were used to predict the 6-week and 12-week changes from baseline). The predictor variables included gut microbiota composition (feature abundances) and predicted functional profiles (KOs pathways). For the outcomes, positive values indicated an increase from the earlier to the later visit, whereas negative values indicated a decrease. The models did not explicitly model within-subject correlation across visits. Longitudinal information was incorporated only through the continuous change scores. Within each subgroup, we applied an unsupervised prevalence filter, retaining features present in more than 10% of samples. Presence was defined as abundance > 0 for genus-level taxa and relative abundance > 5 × 10^–3^ for predicted KEGG level 3 pathways. KEGG features were then CLR-transformed with a small pseudocount as needed. Prevalence filtering retained 56 and 58 genus-level taxa for Phenotypes A and B, respectively, and 51 and 53 Tax4Fun2-predicted KEGG pathways for Phenotypes A and B, respectively. Thus, although the number of retained features remained larger than the phenotype-specific sample sizes, preprocessing reduced the feature dimension to a general order of magnitude with the available subgroup sample sizes, rather than an extremely high-dimensional setting. We applied 3-fold cross-validation across various BART hyperparameter settings (number of trees, node prior, and tree structure priors), selecting the configuration with the lowest mean out-of-sample RMSE. This tuning strategy constrained model complexity through priors that penalize overly deep trees and through evaluation on held-out folds, thereby reducing overfitting risk and favoring configurations with better expected generalization within the current dataset. This configuration was then refit to the full dataset for each subgroup and follow-up time point. Variable importance was summarized by variable inclusion proportions, and model fit was described using in-sample RMSE and RMSE reduction relative to an intercept-only model. The top 10 influential predictors were identified based on their variable inclusion proportions, defined as the proportion of times a variable was used to split a decision node across all trees. The MCMC process included a burn-in period of 10,000 iterations, followed by posterior sampling with every 20th draw retained (thinning), yielding 10,000 posterior samples. Convergence was evaluated using trace plots and autocorrelation functions, confirming adequate mixing and stability of posterior draws.

## 3 Results

### 3.1 Longitudinal multidimensional trajectories-based phenotypes of self-management outcomes in young adults with IBS

Among the 80 participants enrolled in the RCT, 18 contributed only baseline data and could not be assigned to a trajectory cluster (unclassified, baseline-only; n = 18). Of the participants with longitudinal data, 62, who had at least one follow-up, were classified into two multidimensional response trajectories using KML: “Constrained Response Phenotype (Phenotype A, n = 35)” and “Adaptive Multidomain Response Phenotype (Phenotype B, n = 27).” **Table 1** presents the demographic characteristics of participants in Phenotype A and B and those unclassified at baseline. There was no significant difference in demographic characteristics between the two phenotypes and those unclassified at baseline.

**Table 1:** Baseline demographic characteristics.

Characteristic	Phenotype A (n = 35)	Phenotype B (n = 27)	Unclassified (n = 18)	p-value
Age, Mean (SD)	21.83 (2.98)	21.56 (2.38)	20.28 (1.64)	0.105
Year of IBS diagnosis, Mean (SD)	3.49 (3.63)	2.59 (2.21)	3.17 (2.79)	0.517
Sex, n (%)				0.644
Female	25 (71.43%)	22 (81.48%)	14 (77.78%)	
Male	10 (28.57%)	5 (18.52%)	4 (22.22%)	
Race, n (%)				0.195
Asian	5 (14.29%)	4 (14.81%)	1 (5.56%)	
Black or African American	6 (17.14%)	0 (0.0%)	2 (11.11%)	
White	24 (68.57%)	23 (85.19%)	15 (83.33%)	
Ethnicity, n (%)				0.466
Hispanic or Latino	3 (8.57%)	3 (11.11%)	1 (5.56%)	
Not Hispanic or Latino	28 (80.00%)	23 (85.19%)	17 (94.44%)	
Not reported	4 (11.43%)	1 (3.70%)	0 (0.0%)	
Education, n (%)				
High school or below	4 (11.43%)	3 (11.12%)	0	
College or associate degree	18 (51.42%)	12 (44.44%)	16 (88.89%)	
Bachelor’s degree	5 (14.29%)	6 (22.22%)	2 (11.11%)	
Graduate or Doctorate degree	8 (22.86%)	6 (22.22%)	0	
Primary Caregiver, n (%)				
Self-care	16 (45.71%)	16 (59.26%)	8 (44.44%)	
Cared by others	19 (54.29%)	11 (40.74%)	10 (55.56%)	
Employment Status, n (%)				0.159
Student	23 (65.71%)	23 (85.19%)	11 (61.11%)	
Working now	11 (31.43%)	4 (14.81%)	5 (27.78%)	
Unemployed or other	1 (2.86%)	0 (0.0%)	2 (11.11%)	
Marital Status, n (%)				0.584
Never married	2 (5.71%)	1 (3.70%)	0 (0.0%)	
Married	33 (94.29%)	26 (96.30%)	18 (100.00%)	
Family member with IBS, n (%)				0.966
No	24 (68.57%)	19 (70.37%)	12 (66.67%)	
Yes	11 (31.43%)	8 (29.63%)	6 (33.33%)	
Intervention groups, n (%)				0.057
Online Module	23 (65.71%)	12 (44.44%)	6 (33.33%)	
Online Module plus Nurse-led Support	12 (34.29%)	15 (55.56%)	12 (66.67%)	

Phenotype A, Constrained Response Phenotype; Phenotype B, Adaptive Multidomain Response Phenotype; Unclassified, no follow-up data available.

^1^ P-values are to compare the demographic variables between Phenotype A and Phenotype B using the Wilcoxon rank-sum test for the numerical variables and the Fisher’s exact test for the categorical variables.

**Figure 1:**
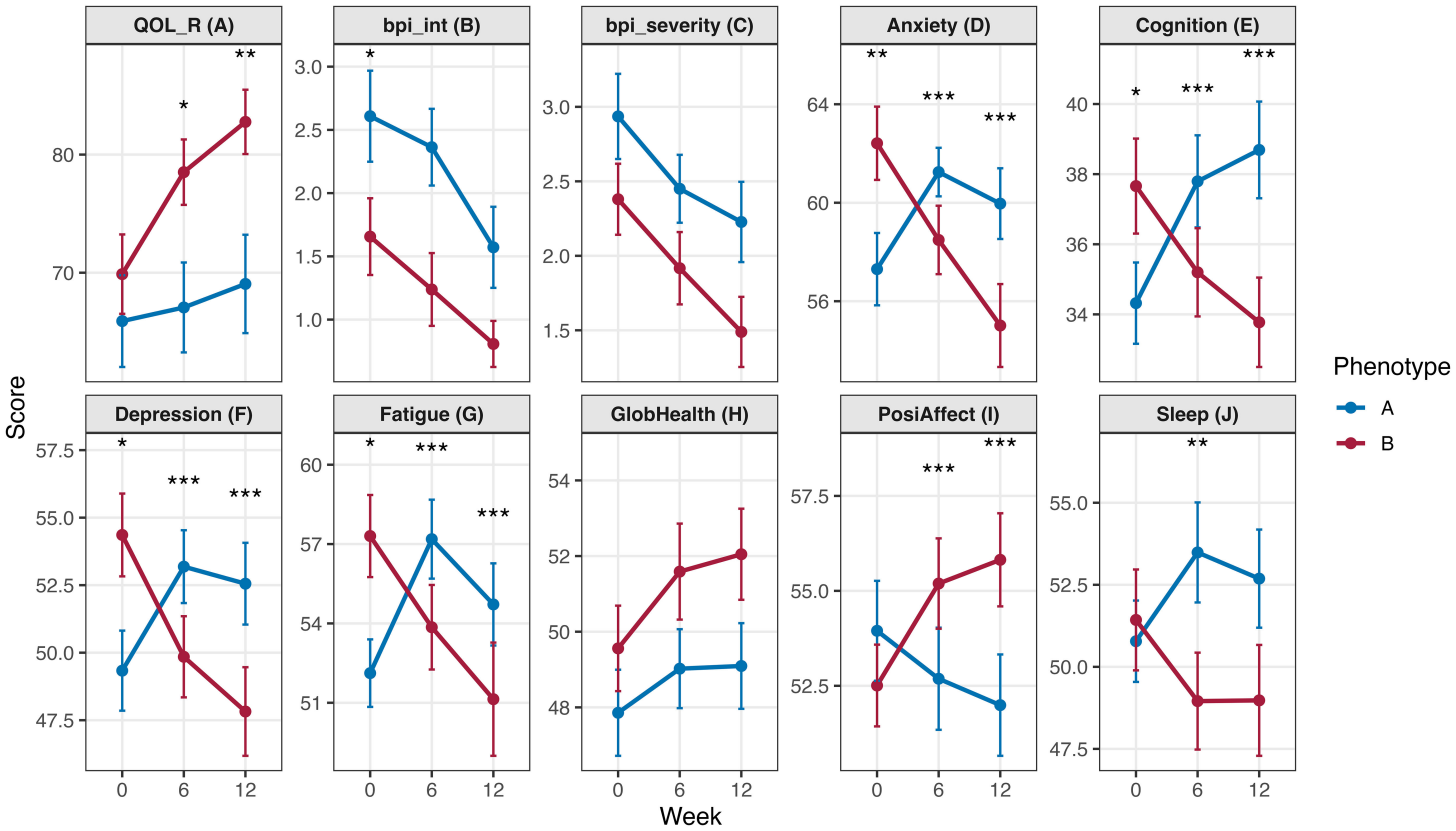
Trajectories of pain, quality of life, and neuropsychological outcomes among the two phenotypes over time. Values shown are adjusted means with standard errors, and p-values are from linear mixed-effects models controlling for sex, race, ethnicity, age, years since IBS diagnosis, and employment status, with a participant-level random intercept. At week 0, p-value annotations represent the adjusted between-phenotype difference at baseline. At weeks 6 and 12, p-value annotations represent the adjusted difference-in-differences comparing changes from baseline between phenotypes. Significance labels are defined as follows: * p < 0.05, ** p < 0.01, and *** p < 0.001. Blue points and lines indicate Phenotype A; red points and lines indicate Phenotype B. **(A)** IBS quality of life; **(B)** BPI pain interference; **(C)** BPI pain severity; and **(D–J)** PROMIS neuropsychological outcomes: anxiety, applied cognition, depression, fatigue, global health, positive affect, and sleep disturbance.

Phenotype A exhibits higher pain levels with limited neuropsychological improvement, while Phenotype B shows lower pain with broad neuropsychological gains. Using Wilcoxon rank-sum tests, Phenotype B had lower BPI interference and severity than Cluster A (p = 0.06 and p = 0.24, respectively), but higher baseline scores in anxiety, depression, and fatigue T-scores (all p < 0.05, **Table 2**). Longitudinal changes from baseline to 12 weeks were assessed with LMM (**Table 3**). Compared to baseline, both phenotypes improved in BPI outcomes at 12 weeks (**Figure 1**, all p < 0.05). Additionally, Phenotype B showed improvements across multiple domains, including better QOL, global health, and positive affect; and reductions in anxiety, applied cognition, depression, fatigue, and sleep disturbance (**Figure 1**, all p < 0.05). Conversely, Phenotype A exhibited more limited improvements, with several domains worsening (e.g., higher anxiety, applied cognition, depression, fatigue, and lower positive affect; all p < 0.05), while QOL saw a modest improvement (p = 0.07). Sleep disturbance showed distinct trajectories, decreasing in Phenotype B and increasing in Phenotype A (p = 0.053).

**Table 2:** Quality of life, pain, and neuropsychological comparison by phenotype across visits. .

	Week 0				Week 0		
Outcome	Phenotype A (N = 35)	PhenotypeB (N = 27)	Not assigned (N = 18)	p-value for Phenotype A vs Phenotype B vs Not assigned	Not assigned (N = 18)	Phenotype A/B combined (N = 62)	p-value for Not assigned vs Phenotype A/B combined
BPI interference	2.61 ± 2.13	1.66 ± 1.58	2.12 ± 1.55	0.128 †	2.12 ± 1.55	2.19 ± 1.95	0.725 †
BPI severity	2.94 ± 1.69	2.38 ± 1.24	2.43 ± 0.99	0.440 †	2.43 ± 0.99	2.69 ± 1.53	0.777 †
Sleep T-score	50.78 ± 7.33	51.43 ± 7.99	51.46 ± 8.88	0.933	51.46 ± 8.88	51.06 ± 7.57	0.851
Fatigue T-score	52.12 ± 7.55	57.30 ± 8.07	58.24 ± 7.76	0.008 †	58.24 ± 7.76	54.38 ± 8.14	0.077
Cognition T-score	34.32 ± 6.86	37.66 ± 7.04	36.59 ± 7.58	0.164 †	36.59 ± 7.58	35.77 ± 7.08	0.665 †
Anxiety T-score	57.30 ± 8.69	62.41 ± 7.73	61.51 ± 9.47	0.051	61.51 ± 9.47	59.53 ± 8.61	0.403
Depression T-score	49.33 ± 8.77	54.36 ± 7.97	50.32 ± 9.72	0.092 †	50.32 ± 9.72	51.52 ± 8.73	0.668 †
Positive affect T-score	53.95 ± 7.78	52.51 ± 5.59	53.88 ± 6.71	0.684	53.88 ± 6.71	53.32 ± 6.90	0.760
Global health T-score	47.86 ± 6.73	49.56 ± 5.87	47.57 ± 7.24	0.508	47.57 ± 7.24	48.60 ± 6.38	0.560

	Week 0			Week 6			Week 12		
Outcome	Phenotype A (N = 35)	Phenotype B (N = 27)	p-value	Phenotype A (N = 35)	Phenotype B (N = 27)	p-value	Phenotype A (N = 33)	Phenotype B (N = 23)	p-value
QOL-R	65.90 ± 22.98	69.88 ± 17.47	0.701 †	67.06 ± 22.52	78.51 ± 14.39	0.050 †	69.05 ± 23.92	82.77 ± 13.06	0.029 †
BPI interference	2.61 ± 2.13	1.66 ± 1.58	0.061 †	2.36 ± 1.79	1.24 ± 1.50	0.007 †	1.57 ± 1.84	0.81 ± 0.87	0.093 †
BPI severity	2.94 ± 1.69	2.38 ± 1.24	0.156	2.45 ± 1.35	1.92 ± 1.26	0.099 †	2.23 ± 1.55	1.49 ± 1.13	0.078 †
Sleep T-score	50.78 ± 7.33	51.43 ± 7.99	0.741	53.49 ± 9.02	48.96 ± 7.67	0.041	52.69 ± 8.59	48.98 ± 8.10	0.033 †
Fatigue T-score	52.12 ± 7.55	57.30 ± 8.07	0.007 †	57.19 ± 8.83	53.86 ± 8.30	0.136	54.72 ± 8.92	51.13 ± 10.27	0.170
Cognition T-score	34.32 ± 6.86	37.66 ± 7.04	0.065	37.79 ± 7.77	35.20 ± 6.54	0.168	38.69 ± 7.92	33.77 ± 6.12	0.016
Anxiety T-score	57.30 ± 8.69	62.41 ± 7.73	0.019	61.25 ± 5.83	58.49 ± 7.22	0.101	59.96 ± 8.25	55.01 ± 8.08	0.030
Depression T-score	49.33 ± 8.77	54.36 ± 7.97	0.023	53.19 ± 7.98	49.85 ± 7.81	0.104	52.55 ± 8.70	47.82 ± 7.87	0.071 †
Positive affect T-score	53.95 ± 7.78	52.51 ± 5.59	0.419	52.69 ± 7.96	55.19 ± 6.18	0.182	51.99 ± 7.67	55.82 ± 5.88	0.049
Global health T-score	47.86 ± 6.73	49.56 ± 5.87	0.301	49.02 ± 6.18	51.59 ± 6.59	0.120	49.09 ± 6.51	52.05 ± 5.77	0.086

Values are presented as mean ± SD. In the first block, baseline outcomes are compared across Phenotype A, Phenotype B, and participants with baseline data only who were not cluster-assigned. P-values compare Phenotype A and Phenotype B within each week using either a two-sample t-test or Wilcoxon rank-sum test, selected according to the Shapiro–Wilk normality assessment. For the three-group baseline comparison, one-way ANOVA or Kruskal–Wallis test was used. † nonparametric tests; otherwise parametric tests.

**Table 3:** Linear mixed-effects model results for quality of life, pain, and neuropsychological outcomes.

Block A									
	QOL_R			BPI interference			BPI severity		
Term	Estimate	SE	p-value	Estimate	SE	p-value	Estimate	SE	p-value
Intercept	51.018	23.571	0.034	5.407	1.811	0.004	5.899	1.425	<0.001
Phenotype B	5.306	5.230	0.314	-1.056	0.442	0.019	-0.652	0.349	0.065
Week 6	1.447	1.924	0.453	-0.238	0.277	0.393	-0.450	0.222	0.045
Week 12	3.556	1.965	0.073	-1.043	0.283	<0.001	-0.646	0.226	0.005
Age	1.211	0.939	0.202	-0.141	0.071	0.052	-0.081	0.056	0.155
Years since IBS diagnosis	0.040	0.647	0.951	-0.079	0.055	0.157	-0.146	0.044	0.001
Female	-11.659	5.138	0.025	0.373	0.430	0.388	-0.298	0.339	0.381
White	-3.162	6.077	0.605	0.075	0.458	0.870	0.063	0.360	0.862
Not Hispanic/Latino	-3.668	8.212	0.657	0.428	0.611	0.487	-0.397	0.480	0.412
Student	3.294	2.840	0.248	-0.229	0.335	0.496	-0.240	0.266	0.368
clusterB:factor(visit)6	7.369	2.915	0.013	-0.159	0.420	0.705	0.032	0.336	0.925
clusterB:factor(visit)12	7.971	3.057	0.010	0.281	0.439	0.523	-0.141	0.351	0.688

**Block B**									
	**Anxiety T-score**			**Cognition T-score**			**Depression T-score**		
**Term**	**Estimate**	**SE**	**p**	**Estimate**	**SE**	**p**	**Estimate**	**SE**	**p**
Intercept	71.247	8.259	<0.001	43.918	8.308	<0.001	59.562	9.660	<0.001
Phenotype B	5.555	1.963	0.006	3.948	1.905	0.041	5.078	2.170	0.022
Week 6	3.830	1.133	<0.001	3.478	0.937	<0.001	3.816	0.925	<0.001
Week 12	2.350	1.156	0.044	4.114	0.956	<0.001	3.057	0.945	0.002
Age	-0.733	0.325	0.028	-0.409	0.329	0.218	-0.564	0.383	0.146
Years since IBS diagnosis	0.473	0.250	0.062	0.353	0.245	0.154	0.322	0.276	0.246
Female	1.858	1.946	0.343	-1.845	1.918	0.339	-0.143	2.176	0.948
White	-0.617	2.094	0.769	-2.249	2.119	0.293	0.914	2.476	0.713
Not Hispanic/Latino	0.523	2.801	0.853	0.439	2.846	0.878	0.634	3.337	0.850
Student	-1.497	1.438	0.299	0.848	1.277	0.508	-0.210	1.320	0.874
clusterB:factor(visit)6	-8.032	1.717	<0.001	-6.003	1.420	<0.001	-8.453	1.402	<0.001
clusterB:factor(visit)12	-9.392	1.796	<0.001	-7.652	1.487	<0.001	-9.523	1.470	<0.001

**Block C**									
	**Fatigue T-score**			**Global Health T-score**			**Positive Affect T-score**		
**Term**	**Estimate**	**SE**	**p**	**Estimate**	**SE**	**p**	**Estimate**	**SE**	**p**
Intercept	75.010	9.679	<0.001	43.170	7.283	<0.001	50.911	8.386	<0.001
Phenotype B	5.403	2.237	0.018	1.596	1.675	0.343	-1.586	1.867	0.398
Week 6	5.108	1.149	<0.001	1.133	0.837	0.179	-1.286	0.723	0.078
Week 12	2.411	1.173	0.042	1.091	0.855	0.204	-1.929	0.739	0.010
Age	-0.862	0.382	0.028	0.056	0.288	0.847	0.222	0.334	0.509
Years since IBS diagnosis	0.099	0.288	0.731	0.260	0.215	0.230	-0.042	0.234	0.857
Female	-1.210	2.248	0.592	-0.460	1.685	0.786	0.858	1.851	0.644
White	-3.641	2.464	0.145	3.117	1.856	0.099	-2.551	2.158	0.242
Not Hispanic/Latino	-2.286	3.307	0.492	1.145	2.492	0.648	-2.225	2.913	0.448
Student	1.555	1.540	0.314	-0.455	1.134	0.689	2.307	1.057	0.030
clusterB:factor(visit)6	-8.449	1.742	<0.001	0.755	1.269	0.553	4.152	1.096	<0.001
clusterB:factor(visit)12	-7.578	1.824	<0.001	1.732	1.329	0.195	6.035	1.150	<0.001

**Block D**									
	**Sleep T-score**								
**Term**	**Estimate**	**SE**	**p-value**						
Age	-0.967	0.356	0.009						
Years since IBS diagnosis	-0.047	0.271	0.862						
Female	0.632	2.112	0.765						
White	-3.962	2.292	0.089						
Not Hispanic/Latino	0.312	3.071	0.919						
Student	-1.572	1.504	0.298						
clusterB:factor(visit)6	-5.259	1.747	0.003						
clusterB:factor(visit)12	-3.567	1.828	0.053						

Separate linear mixed-effects models were fitted for each outcome. Fixed effects included week, phenotype, their interaction, and covariates (age, years since IBS diagnosis, sex, race, Hispanic/Latino ethnicity, and student status); participant was included as a random intercept. Week 0 and Phenotype A were used as the reference categories.

### 3.2 Biological differences in gut microbiota composition and function among distinct IBS phenotypes

**Figure 2:**
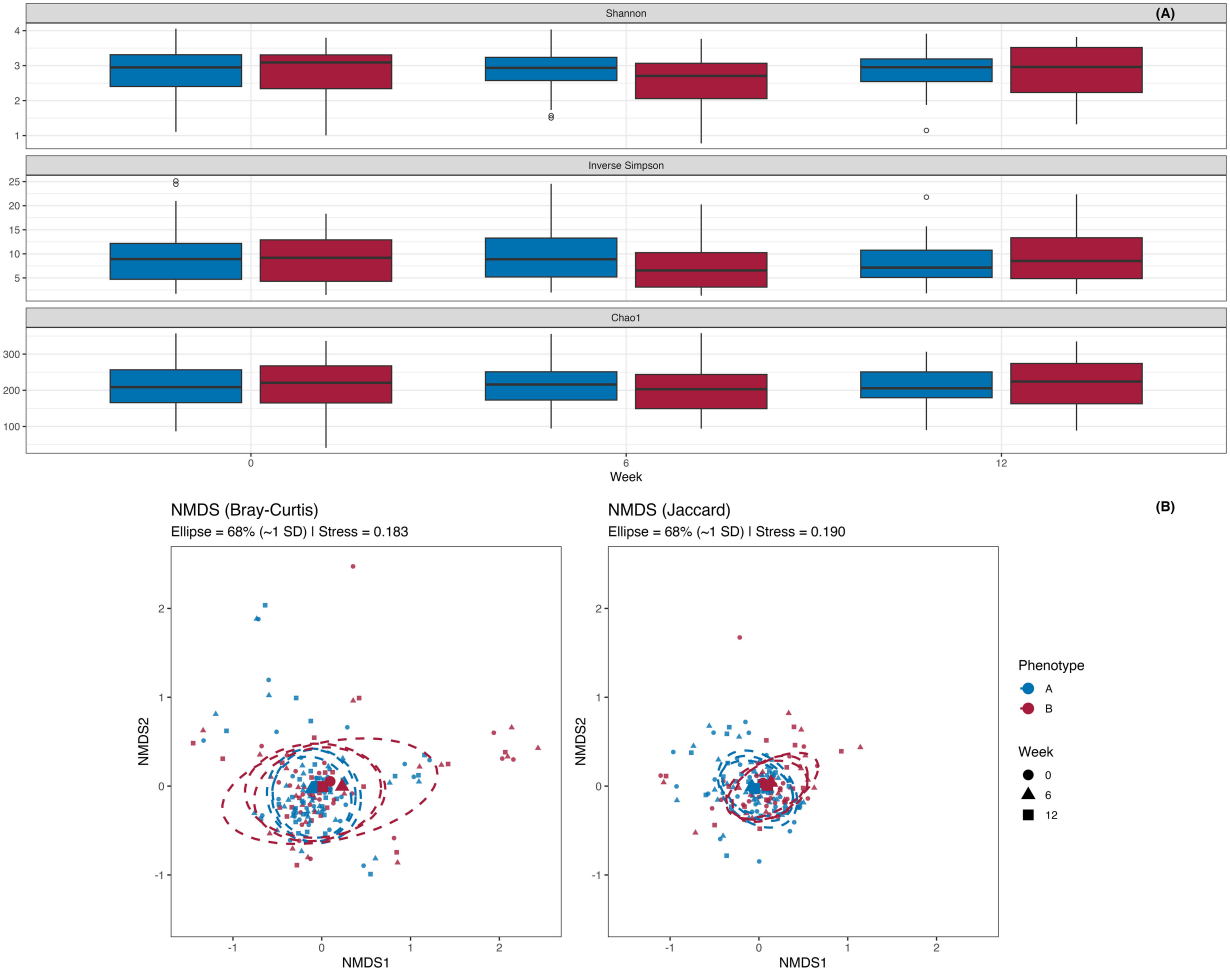
Diversity of the bacterial community among the two phenotypes over time. **(A)** a-diversity trajectories among the two phenotypes over time. After fitting a linear mixed-effects model (LMM) adjusting for sex, race, ethnicity, age, years since IBS diagnosis, and employment status, Inverse Simpson diversity in Phenotype A showed a significant decrease at week 12 (β = −1.546, p = 0.039). No statistically significant week-12 changes were observed for Shannon or Chao1. A1. Shannon index. A2. Inverse Simpson index. A3. Chao index. **(B)** β-diversity trajectories among the two phenotypes over time. By PERMANOVA (adonis2; 999 permutations; marginal tests), Bray–Curtis beta diversity was not significantly associated with phenotype at baseline (R² = 0.023, p = 0.110), but was significantly associated with phenotype at week 6 (R² = 0.044, p = 0.007) and week 12 (R² = 0.030, p = 0.028), after adjusting for sex, race, ethnicity, employment status, age, and years since IBS diagnosis. Across timepoints, race was consistently associated with community composition (baseline p = 0.001; week 6 p = 0.006; week 12 p = 0.001), and years since IBS diagnosis was also associated at baseline (p = 0.032) and week 12 (p = 0.008). B1. Bray-Curtis dissimilarity; B2. Jaccard dissimilarity.

The unadjusted comparisons showed no statistically significant differences in alpha-diversity trajectories within any phenotypes (**Figure 2A**; **Table 4**). After fitting a linear mixed-effects model (LMM) adjusting for sex, race, ethnicity, age, years since IBS diagnosis, and employment status, Inverse Simpson diversity in Phenotype A showed a significant decrease at week 12 (β = −1.546, p = 0.039). No statistically significant week-12 changes were observed for Shannon or Chao1.

**Table 4:** Linear mixed-effects model results for alpha-diversity indices.

	Shannon			Inverse Simpson			Chao1		
Term	Estimate	SE	p-value	Estimate	SE	p-value	Estimate	SE	p-value
Intercept	1.724	0.802	0.036	3.786	6.015	0.531	134.859	74.304	0.074
Week 6	0.039	0.077	0.613	0.014	0.732	0.985	2.657	7.457	0.722
Week 12	-0.045	0.078	0.562	-1.546	0.741	0.039	3.838	7.546	0.612
Phenotype B	-0.292	0.180	0.109	-1.756	1.388	0.209	-10.844	16.729	0.519
Female	0.124	0.179	0.490	1.183	1.389	0.397	20.499	16.715	0.223
White	0.796	0.206	<0.001	5.303	1.529	0.001	66.431	19.054	<0.001
Not Hispanic/Latino	-0.101	0.276	0.715	-1.624	2.034	0.428	-4.960	25.448	0.846
Age	0.036	0.032	0.268	0.176	0.240	0.464	2.030	2.967	0.496
Student	-0.089	0.107	0.405	-1.148	0.951	0.229	-21.399	10.240	0.038
Years since IBS diagnosis	-0.029	0.027	0.281	-0.034	0.197	0.863	-3.558	2.465	0.155
Week 6 × Phenotype B	-0.146	0.120	0.226	-1.117	1.139	0.329	-1.973	11.613	0.865
Week 12 × Phenotype B	0.076	0.121	0.532	1.845	1.153	0.112	-0.444	11.745	0.970

Separate linear mixed-effects models were fitted for Shannon, inverse Simpson, and Chao1 indices. Fixed effects included week, phenotype, their interaction, and covariates; participant was included as a random intercept. Week 0 and Phenotype A were used as the reference categories.

In a repeated-measures PERMANOVA with permutations constrained within participant, the visit-by-phenotype interaction was also not significant (**Figure 2B**; **Table 5**). Bray–Curtis beta diversity was not significantly associated with phenotypes at baseline (R² = 0.023, p = 0.110), but was significantly associated with cluster at week 6 (R² = 0.044, p = 0.007) and week 12 (R² = 0.030, p = 0.028), after adjusting for sex, race, ethnicity, employment status, age, and years since IBS diagnosis. Across timepoints, race was consistently associated with community composition (baseline p = 0.001; week 6 p = 0.006; week 12 p = 0.001), and years since IBS diagnosis was also associated at baseline (p = 0.032) and week 12 (p = 0.008).

**Table 5:** PERMANOVA results for beta diversity by distance metric.

		Week 0		Week 6		Week 12		Repeated	
Measure	Predictor	Marginal R²	p-value	Marginal R²	p-value	Marginal R²	p-value	Marginal R²	p-value
Bray-Curtis	Phenotype	0.023	0.110	0.044	0.007	0.030	0.028		
	Female	0.019	0.231	0.019	0.330	0.019	0.306	0.016	0.511
	White	0.074	0.001	0.043	0.006	0.068	0.001	0.058	0.928
	Not Hispanic/Latino	0.011	0.780	0.014	0.672	0.021	0.219	0.012	0.124
	Student	0.017	0.324	0.016	0.482	0.015	0.554	0.009	0.275
	Age	0.008	0.955	0.010	0.887	0.019	0.336	0.008	0.363
	Years since IBS diagnosis	0.029	0.032	0.026	0.112	0.040	0.008	0.029	0.397
	Week × Phenotype							0.005	0.138
Jaccard	Phenotype	0.020	0.188	0.034	0.010	0.028	0.018		
	Female	0.016	0.416	0.017	0.477	0.018	0.385	0.012	0.532
	White	0.055	0.001	0.034	0.007	0.052	0.001	0.042	0.923
	Not Hispanic/Latino	0.012	0.835	0.015	0.699	0.020	0.258	0.011	0.231
	Student	0.016	0.419	0.016	0.541	0.017	0.488	0.009	0.261
	Age	0.011	0.945	0.012	0.948	0.019	0.318	0.008	0.404
	Years since IBS diagnosis	0.026	0.028	0.024	0.111	0.034	0.008	0.024	0.458
	Week × Phenotype							0.006	0.076

Marginal R² and permutation p-values are shown for PERMANOVA models using 999 permutations. Cross-sectional analyses were performed separately at Week 0, Week 6, and Week 12. Repeated-measures analyses included week, phenotype, their interaction, and covariates, with permutations constrained within participant.

**Figure 3:**
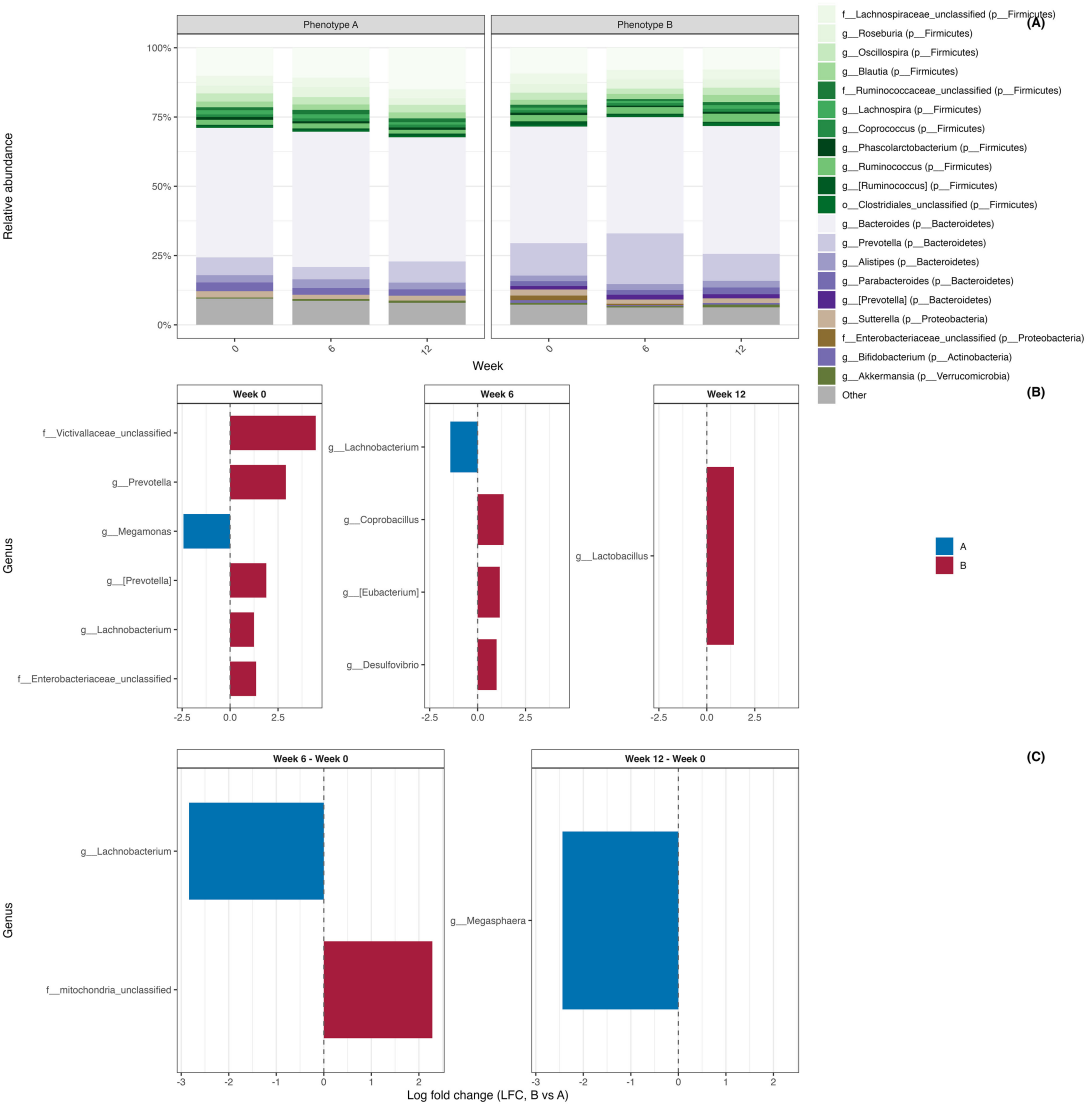
Gut microbiota compositional differences between the two phenotypes over time. **(A)** Relative abundance of dominant genera (colored by phylum) across Week 0, Week 6, and Week 12 for Phenotype A and Phenotype B Stacked bar plots represent mean compositional profiles within each phenotype and time point. **(B)** Differential abundance analysis at each time point, shown as log fold change (LFC, Phenotype B vs. Phenotype A). Positive values indicate higher abundance in Phenotype B, while negative values indicate higher abundance in Phenotype A The Holm procedure was used to control the false discovery rate. Only taxa with significant differences are displayed. **(C)** Longitudinal changes in differential abundance, represented as differences in LFC between time points (Week 6 − Week 0 and Week 12 − Week 0). These panels highlight taxa that exhibit phenotype-specific temporal shifts (i.e., interactions between phenotype and time). **(A)** (two phenotypes over time, baseline, 6 weeks, 12 weeks) bar plot. B1. difference at baseline. B2. at week 6. B3. week 12. C1. Interaction effect at week 6. C2. Interaction effect at week 12.

Exploratory analyses of longitudinal gut microbiota features among participants with available stool samples across the two phenotypes showed a compositional shift from baseline to week 12 (**Figure 3**). Exploratory genus-level differential abundance analyses using ANCOM-BC2 suggested between-cluster differences in trajectories (**Figure 3**). *Prevotella* and *Lachnobacterium* showed an estimated increase in abundance in Phenotype B, and *Megamonas* was increased in abundance in Phenotype A at baseline (**Figure 3B1**). At week 6 follow-up, the abundances of four genera were significantly different between the two phenotypes, with higher enrichment of *Lachnobacterium* in Phenotype A and higher enrichment of *Desulfovibrio*, *Coprobacillus*, and *Eubacterium* in Phenotype B (**Figure 3B2**). *Lactobacillus* showed an estimated increase in abundance in Phenotype B at week 12 (**Figure 3B3**). Moreover, the abundance of three genera shows compositional differences over time and across phenotypes (**Figure 3C**). Compared to Phenotype A, Phenotype B showed an estimated decrease in *Lachnobacterium* abundance and increased abundance of *Mitochondria-unclassified* at week 6, and a decrease in *Megasphaera* abundance at week 12.

**Figure 4:**
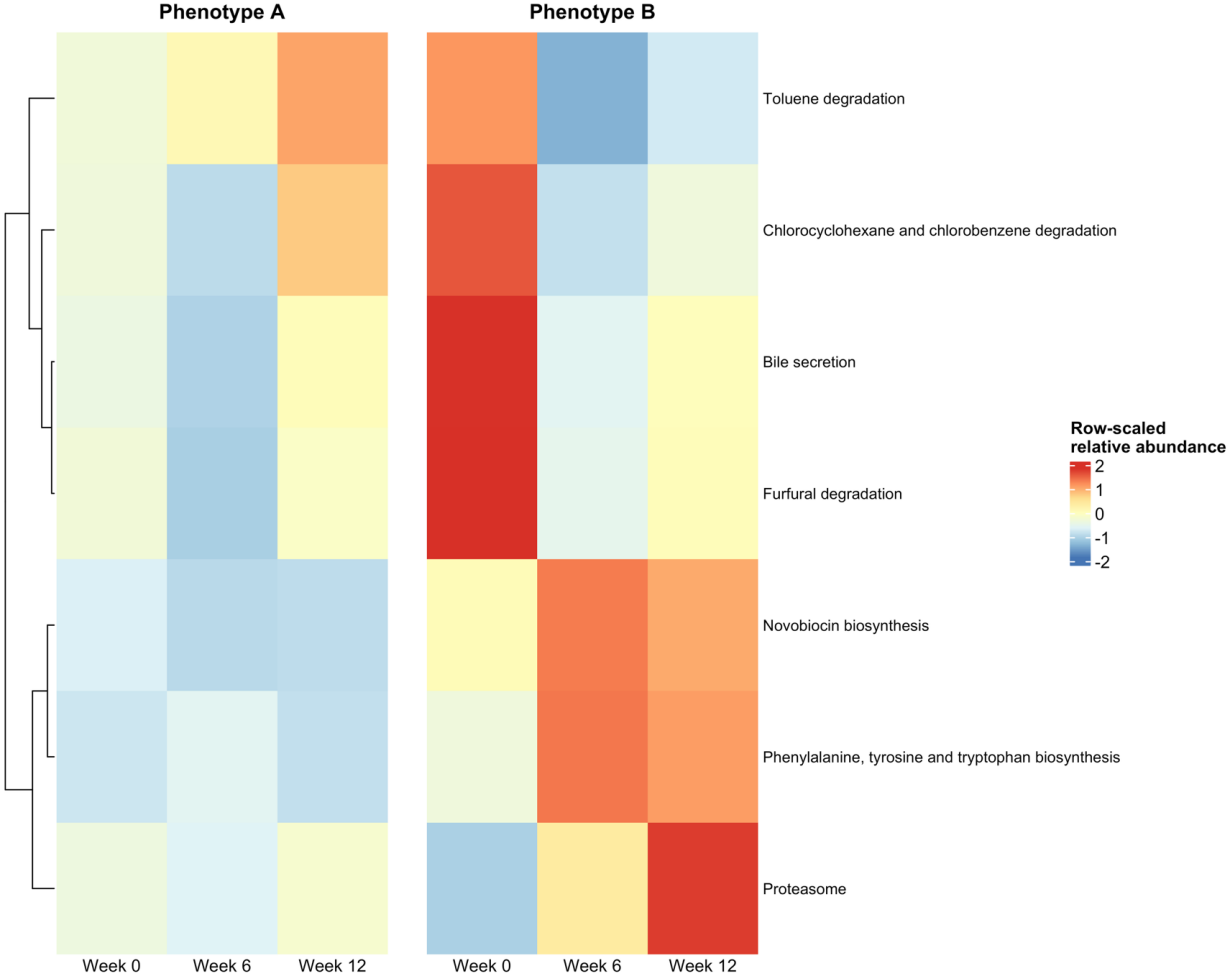
Different metabolic profiles of gut microbiota among the two phenotypes over time. Heatmap of pathway-level functional profiles across visits and phenotypes. Rows show the top pathways selected by unadjusted significance (raw p-values), and columns show the phenotype by visits combinations. Each cell represents the mean relative abundance of a pathway within that phenotype and visit, standardized within each pathway (row-wise z-score) to highlight patterns rather than absolute abundance. Warmer colors indicate time points where a pathway is above its own average level across all columns, and cooler colors indicate below its own average level. Negative values therefore reflect below-average relative abundance. Differences in KEGG pathways (level 3) were assessed using linear models with empirical Bayes moderation. The Benjamini-Hochberg procedure was used to control the false discovery rate for multiple test corrections. The p-value was set as 0.05. 7 microbial pathways differed between two clusters (raw p<0.05); none survived FDR.

Using limma, we identified seven predicted metabolic pathways derived from gut microbiota differences in change from baseline to week 12 for KEGG functional pathways (raw p < 0.05; **Figure 4**), including xenobiotic degradation (ko00361/ko00623/ko00365), aromatic amino acid biosynthesis (ko00400), bile secretion (ko04976), proteasome (ko03050), and novobiocin biosynthesis (ko00401), suggesting heterogeneous functional dynamics by symptom-trajectory phenotype; although none remained significant after FDR correction. As shown in the row-scaled heatmap of the top unadjusted pathways across weeks 0, 6, and 12 (**Figure 4**), pathway-level changes were heterogeneous. Some pathways exhibited broadly parallel warm-to-cool shifts from baseline to week 12 in both phenotypes, while others showed transient, time-dependent fluctuations without a consistent intervention-specific separation. After adjusting for multiple testing, no pathway reached FDR significance (minimum adjusted p = 0.40); in both phenotypes, prodigiosin biosynthesis and the Caulobacter cell cycle decreased from baseline to week 12 (unadjusted p < 0.05; FDR-adjusted p > 0.05).

### 3.3 Gut microbiota can predict the outcomes of IBS pain self-management interventions

**Figure 5:**
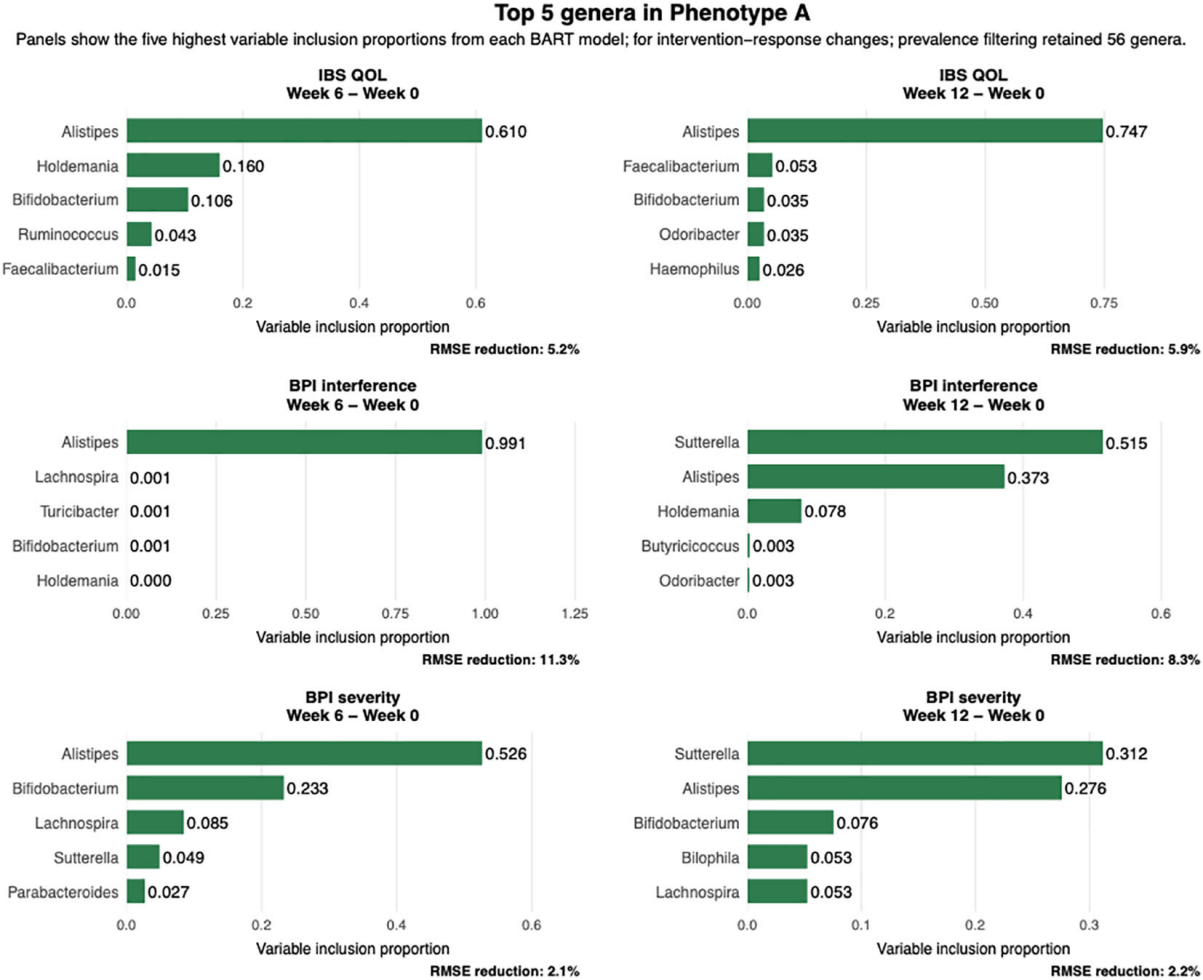
The top 5 genera of baseline gut microbiota predict intervention response, defined as changes in pain severity, pain interference, and quality of life at 6 and 12 weeks in Phenotype A.

**Figure 6:**
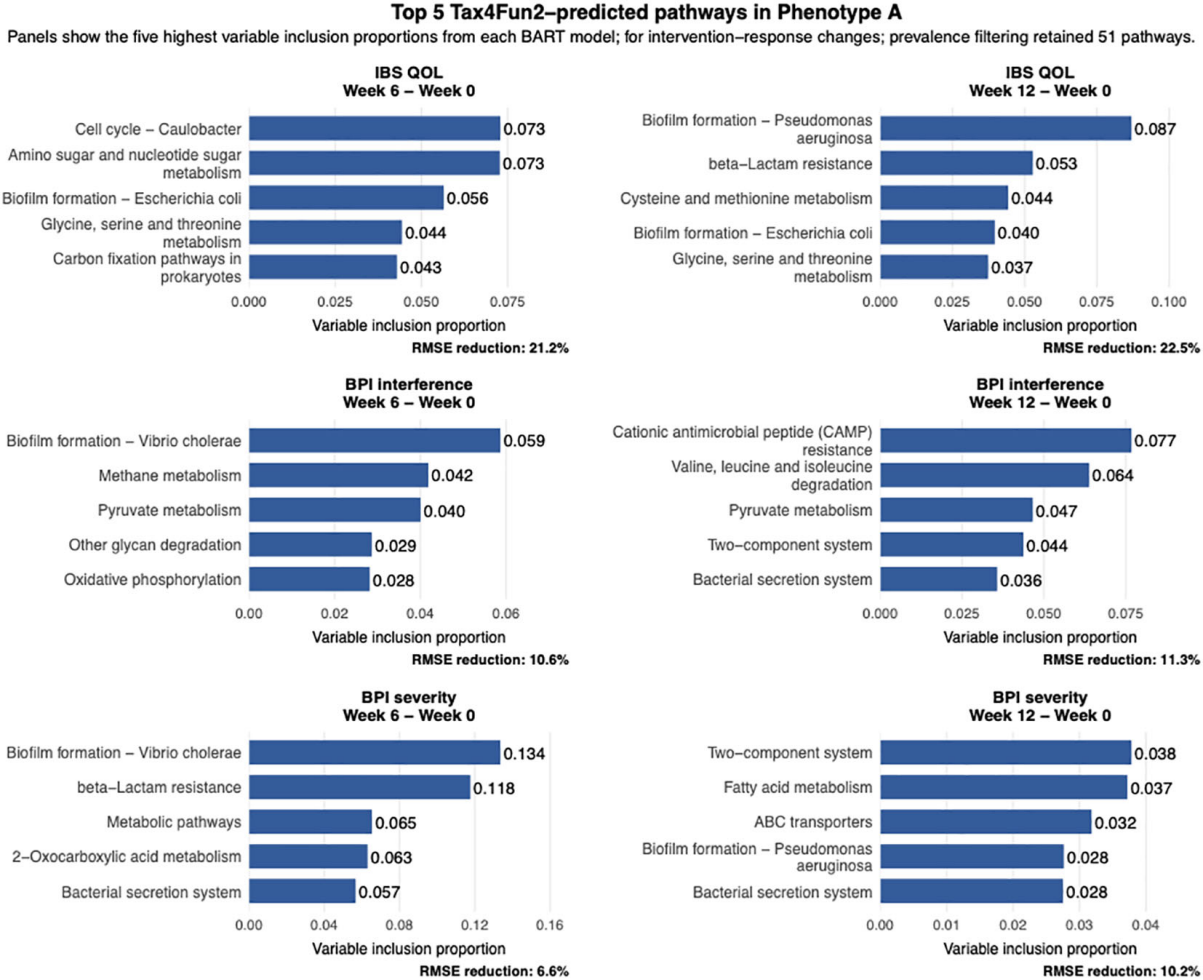
The top 5 Tax4Fun2-predicted pathways of baseline gut microbiota predict intervention response, defined as changes in pain severity, pain interference, and quality of life at 6 and 12 weeks in Phenotype A.

**Figure 7:**
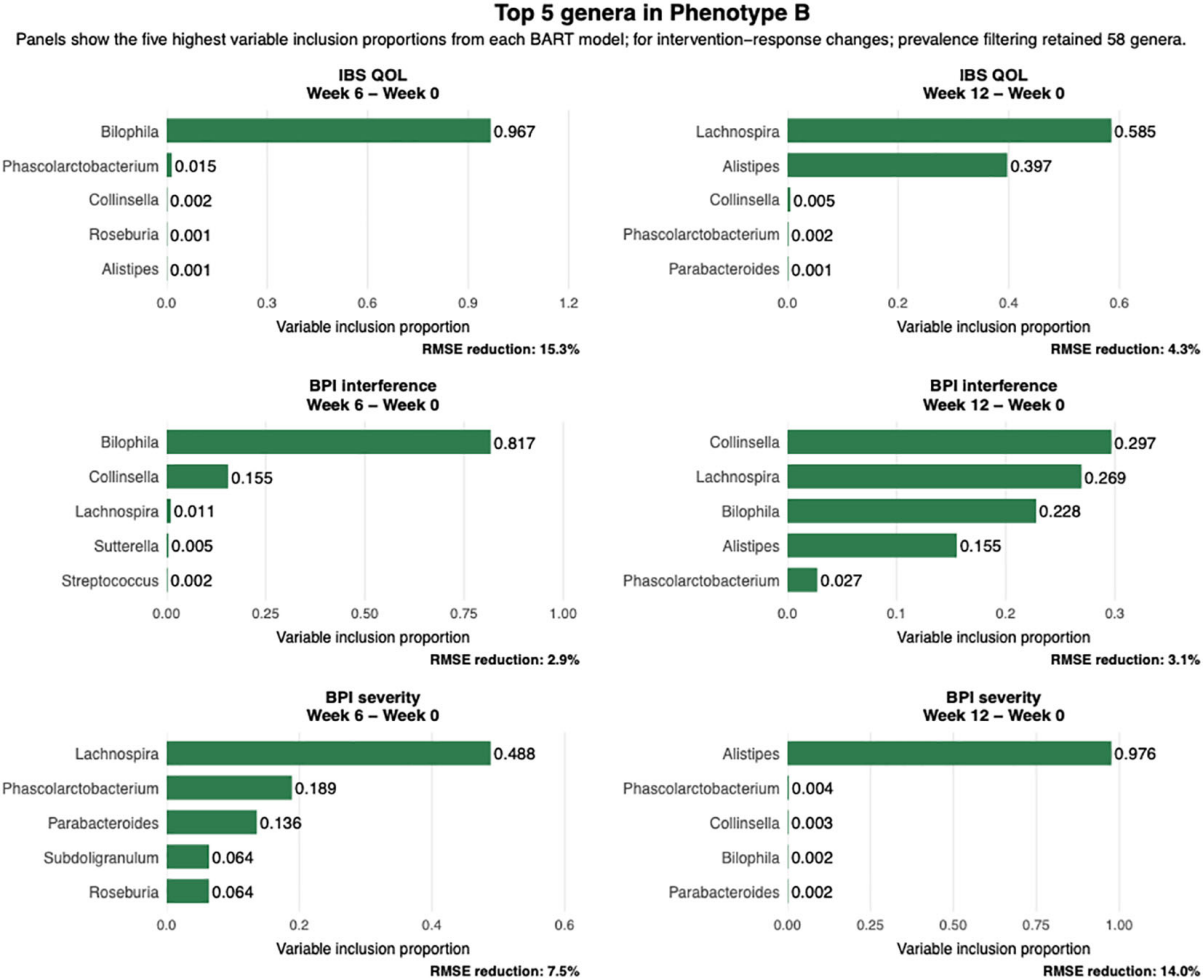
The top 5 genera of baseline gut microbiota predict intervention response, defined as changes in pain severity, pain interference, and quality of life at 6 and 12 weeks in Phenotype B.

**Figure 8:**
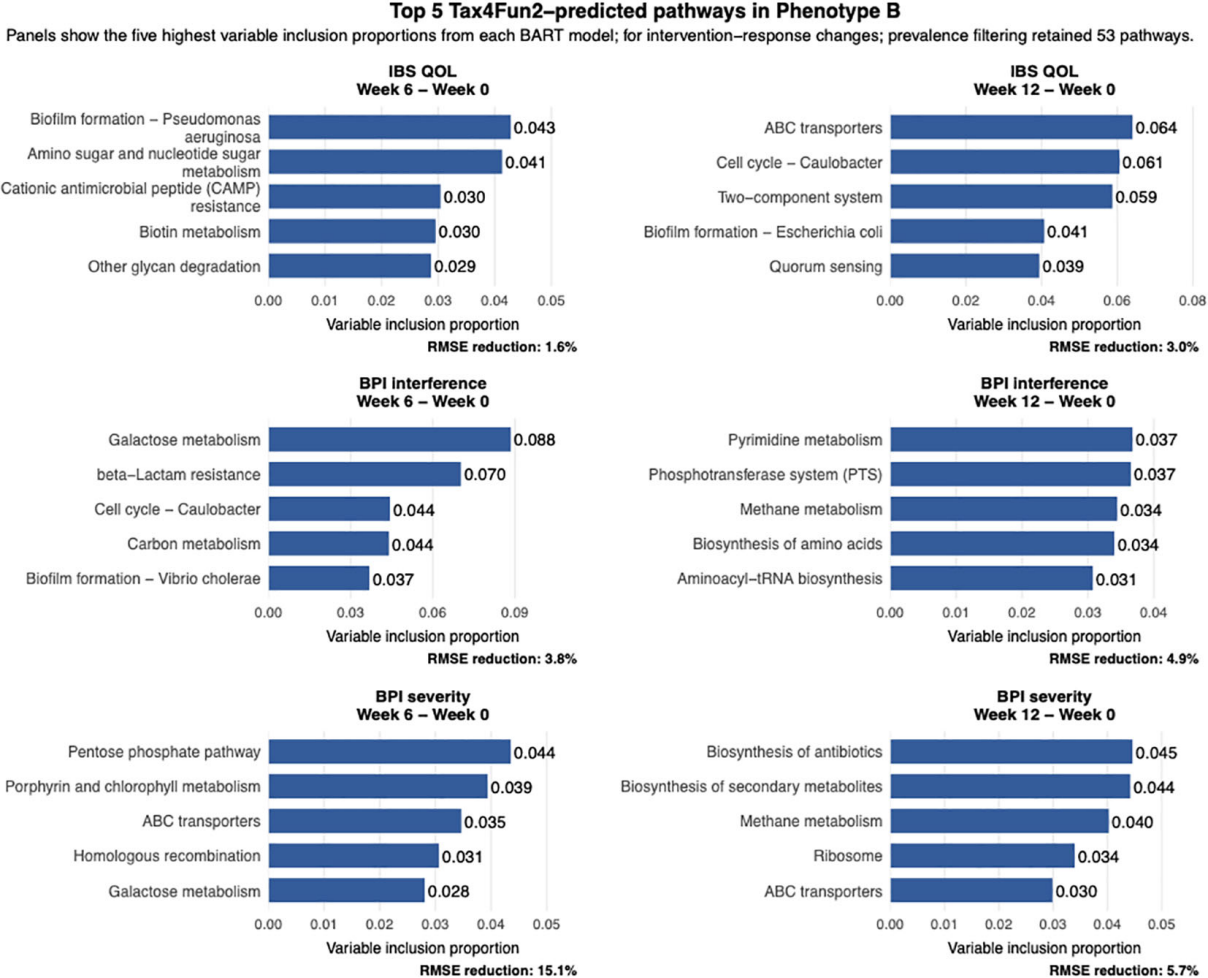
The top 5 Tax4Fun2-predicted pathways of baseline gut microbiota predict intervention response, defined as changes in pain severity, pain interference, and quality of life at 6 and 12 weeks in Phenotype B.

BART models were applied to identify baseline gut microbial genera and functional pathways (KEGG Level 3) that serve as influential predictors of intervention response, defined as changes in pain severity, pain interference, and quality of life at 6 and 12 weeks. BART models were developed using baseline gut microbial genera and Tax4Fun2-predicted microbial pathways for Phenotypes A and B (**Figures 5**–**8**; **Supplementary Figures 1**–**4**) including three prediction models for change from week 0 to week 6 outcomes (pain severity, pain interference, and quality of life) and three corresponding models for week 12 outcomes. Phenotype-specific models (Phenotype A and Phenotype B) were used to characterize distinct microbiome–phenotype response profiles. To address sparsity and zero inflation in high-dimensional microbiome data, a prevalence-based filtering step was applied within each cluster, retaining features present in >10% of samples. For genus-level taxa, presence was defined as abundance >0, whereas for Tax4Fun2-predicted KEGG pathways, presence was defined using a minimum relative abundance threshold (5e-3). The RMSEs of these 12 models ranged from 8.011 to 13.965 for QOL and from 0.858 to 1.714 for pain (**Table 6**), indicating moderate discriminative ability. Across models, full-sample RMSEs were slightly lower than cross-validated out-of-sample RMSEs, and in-sample estimates closely approximated full-sample performance (**Table 6**), suggesting minimal overfitting.

**Table 6:** RMSE summary by feature type and phenotype based on the BART models.

Model	Outcome	Week 6 - week 0	Week 12 - week 0
Genus - Phenotype A	QOL_R	9.212 (8.825, 10.776)	13.965 (14.890, 15.745)
	bpi_int	1.660 (1.765, 2.153)	1.714 (1.768, 1.951)
	bpi_severity	1.274 (1.240, 1.372)	1.457 (1.481, 1.567)
Genus - Phenotype B	QOL_R	8.011 (8.814, 8.687)	9.741 (9.734, 9.982)
	bpi_int	1.164 (1.167, 1.244)	1.168 (1.185, 1.296)
	bpi_severity	0.858 (0.898, 0.915)	1.025 (1.098, 1.276)
Pathway - Phenotype A	QOL_R	7.655 (8.784, 9.769)	11.503 (12.868, 14.880)
	bpi_int	1.675 (1.760, 2.005)	1.658 (1.685, 1.829)
	bpi_severity	1.215 (1.247, 1.374)	1.338 (1.349, 1.696)
Pathway - Phenotype B	QOL_R	9.306 (9.401, 10.104)	9.870 (10.112, 10.351)
	bpi_int	1.154 (1.136, 1.229)	1.147 (1.115, 1.317)
	bpi_severity	0.787 (0.846, 0.913)	1.123 (1.125, 1.411)

3-fold cross-validation was used to select the best hyperparameter configuration for each outcome-task combination (row) based on mean out-of-sample RMSE across visits (columns). The selected configuration was then applied to the full dataset to obtain the final RMSE. Each cell shows the full-data RMSE, with mean in-sample and out-of-sample RMSEs in parentheses.

The top 5 baseline gut microbial genera identified by variable importance measures varied across models and phenotypes (**Figures 5**, **7**). In Phenotype A, *Alistipes* and *Sutterella* were consistently present across all models, indicating robust and reproducible microbiome predictors of intervention response; *Bifidobacterium* and *Ruminoccoccus* were also included in most models (**Figure 5**; **Supplementary Figure 1**). In Phenotype B, *Phascolarctobacterium* was consistently present in all models, followed by *Alistipes*, *Collinsella*, and *Parabacteroides*, each present in 5 models (**Figure 7**; **Supplementary Figure 2**).

The top 5 baseline gut microbiota pathways that predicted intervention response also differed across phenotypes and visits (**Figures 6**, **8**; **Supplementary Figures 3**, **4**). In Phenotype A, the pathways “Glycine, serine and threonine metabolism” and “Cysteine and methionine metabolism” were consistently among the predictors of changes in QOL from baseline to follow-ups; “Pyruvate metabolism” consistently predicted changes in BPI pain interference; and “Bacterial secretion system” consistently predicted changes in BPI severity (**Figure 6**; **Supplementary Figure 3**). Similar but distinct patterns in Phenotype B: only “Porphyrin and chlorophyll metabolism” was consistently among the predictors of changes in QOL from baseline to follow-ups; “Butanoate metabolism” consistently predicted changes in BPI pain interference; “Mismatch repair” consistently predicted changes in BPI severity (**Figure 8**; **Supplementary Figure 4**).

## 4 Discussion

This analysis identified phenotype-specific mechanisms of the gut microbiome involved in pain self-management programs in young adults with IBS. Although both interventions were effective in improving patient-centered outcomes (Chen et al., 2022), patients did not respond uniformly; instead, they segregated into distinct multidimensional response phenotypes that were partially independent of treatment assignment. These findings indicate that intervention effects unfold within heterogeneous regulatory contexts, characterized by distinct neuropsychological and microbiome-linked response trajectories. While primary analyses demonstrated overall intervention efficacy, clustering revealed substantial heterogeneity, identifying phenotypes that may reflect underlying differences in regulatory capacity and gut–brain axis function (Khan and Chang, 2010; Ford et al., 2017; Ford et al., 2020).

The current analysis does not establish causal relationships between baseline microbiota and subsequent clinical response. The BART models were designed to identify baseline microbial features associated with, and predictive of, 12-week changes in clinical outcomes, rather than to infer causal mechanisms. Building on this, our findings show that gut microbiota were not only associated with variability in outcomes but also with phenotype-specific predictive signatures. Baseline microbial genera were used to predict distinct microbiome-phenotype response profiles, suggesting that the microbial community may drive variation in responses to interventions and may be used to inform optimal interventions at the individual level. In Phenotype A, genera such as *Alistipes* and *Sutterella* were consistently identified across all six models (**Figure 5**; **Supplementary Figure 1**), with *Bifidobacterium, Lachnospira*, and *Ruminococcus* also frequently contributing to the changes in BPI severity, indicating a relatively stable and reproducible microbial signature. *Alistipes* and *Sutterella* were identified as important predictors in BART models in Phenotype A, although the biological mechanisms linking these taxa to treatment response remain unclear in patients with IBS. Increased *Alistipes* has been identified as pathogenic in colorectal cancer and is linked to the risk of depression (Parker et al., 2020), while *Bifidobacterium* is thought to offer health benefits (O’Callaghan and van Sinderen, 2016). Previous studies have confirmed that higher levels of *Ruminococcus* and *Sutterella* are associated with increased IBS pain and a greater risk of cognitive decline (Hollister et al., 2020; Meyer et al., 2022; Wei et al., 2025). These abundance features align with the symptom trajectory in Phenotype A, which shows improvements in pain but worsening in several neuropsychological domains at 6 and 12 weeks, such as depression and applied cognition (**Figure 1**).

In contrast, Phenotype B was characterized by a different set of predictors, with *Phascolarctobacterium* consistently present and *Alistipes*, *Collinsella*, and *Parabacteroides* appearing in most models (**Figure 7**; **Supplementary Figure 2**). Although the RMSE of *Phascolarctobacterium, Collinsella*, and *Parabacteroides* in the BART models was small, previous studies have confirmed their health benefits in IBS, including reducing inflammation, improving intestinal barrier integrity, and modulating the microbiota-gut-brain axis (Gargari et al., 2024; Lan et al., 2025; Liébana-García et al., 2025; Li et al., 2025). This divergence suggests that different microbial arrangements may lead to similar clinical improvements, indicating multiple biological pathways that result in comparable symptom outcomes. The phenotype-response may guide precision health strategies for IBS self-management by targeting gut microbiomes underlying the condition.

Although there were nominal differences in predicted functional pathway profiles between phenotypes, these differences were not significant after FDR correction. However, when using baseline-predicted microbial metabolic pathways to forecast phenotype response profiles, a similar pattern was seen at the functional level in the BART models, with predicted microbial metabolic pathways showing variation across phenotypes and outcomes. In Phenotype A, pathways related to amino acid metabolism (e.g., glycine, serine, threonine; cysteine and methionine) were consistently associated with improvements in quality of life, while pyruvate metabolism and bacterial secretion systems were linked to changes in pain interference and severity, respectively (**Figure 6**; **Supplementary Figure 3**). These functional pathways regulate tryptophan metabolism, an essential amino acid, thereby affecting host physiology and immunity, linking microbial ecology with neuroimmune signaling and gut–brain interactions (Agus et al., 2018; Kaur et al., 2019). In contrast, Phenotype B demonstrated a distinct functional profile, with pathways such as porphyrin metabolism, butanoate metabolism, and mismatch repair emerging as consistent predictors of quality of life and pain outcomes (**Figure 8**; **Supplementary Figure 4**). These findings suggest that functional capacity of the microbiome, rather than taxonomic composition alone, may differentially shape symptom trajectories across phenotypes, potentially through effects on host metabolic, immune, and neuroregulatory processes.

Together, these results extend prior work by demonstrating that microbiome-associated mechanisms of treatment response are not uniform but instead operate through phenotype-specific microbial and functional pathways. While pathway-level differences did not remain statistically significant after multiple testing correction, their consistency across models, alignment with known IBS-related biological processes, and convergence at both taxonomic and functional levels support their biological plausibility (Jacobs et al., 2023; Chen et al., 2024; Wu et al., 2025). These findings highlight the importance of integrating machine learning–derived feature selection with theory-driven interpretation to uncover meaningful biological signals in high-dimensional microbiome data. Our findings suggest that microbial functional profiles may be more informative as multivariable predictors of treatment response than as individually differentially abundant pathways. However, because pathway predictions were inferred from 16S rRNA data and the BART findings were exploratory, they should be interpreted as hypothesis-generating rather than confirmatory.

The predictive performance of machine learning models provides further support for the role of the gut microbiome in regulating IBS symptoms. Baseline microbial features were informative in predicting both symptom severity and longitudinal changes, suggesting that microbiome profiles may serve as candidate biomarkers for stratifying patients and tailoring self-management strategies. Importantly, the variability in selected features across phenotypes underscores the need for precision approaches that account for underlying heterogeneity rather than assuming a single uniform microbiome signature of response.

These findings align with emerging multi-omics evidence demonstrating coordinated alterations in gut microbial function and host biological systems in IBS (Major and Spiller, 2014; Bhattarai et al., 2017; Mayer et al., 2023). Within this framework, self-management interventions may act as modulators of a broader regulatory network linking microbial metabolism, host immune activity, and neuroendocrine function. The observed phenotype-specific microbiome signatures further suggest that intervention effects may be mediated through different regulatory pathways across individuals, consistent with a systems-based model of gut–brain axis dysregulation.

Baseline microbial features predicted 12-week clinical response in a phenotype-specific manner; however, these findings do not imply causality. The taxa identified by BART should be interpreted as predictive biomarkers rather than mechanistic drivers. Moreover, distinct microbial taxa and predicted functional features were associated with response phenotypes; the functional pathway findings should be considered hypothesis-generating and require validation in larger cohorts using direct functional profiling approaches. Further studies integrating longitudinal microbial profiling, microbial metabolites, host immune and neuroendocrine measures, and experimental validation are needed to clarify the biological roles of these taxa in IBS symptom improvement.

### 4.1 Limitations

Several limitations should be considered. First, although no significant differences were observed in baseline demographic characteristics between groups, potentially important confounding factors were not measured. IBS subtype and sex hormone-related variables were not collected. These factors may influence symptom presentation, disease mechanisms, and gut microbiota composition. Second, disease duration and medication use were not comprehensively assessed, which may have contributed to inter-individual variability in microbiome profiles and clinical outcomes. Future studies should incorporate these variables to better elucidate the complex relationships among clinical phenotypes, host factors, and the gut microbiome. Third, the sample size was modest, reducing statistical power to detect pathway-level differences after multiple-comparison correction and limiting generalizability. Because the present study focused on prediction rather than treatment efficacy, the findings should be interpreted as identifying candidate microbial markers associated with clinically meaningful response patterns previously demonstrated in the parent RCT.

Another limitation of this study is that microbial functional profiles were inferred from 16S rRNA sequencing data using Tax4Fun2 rather than directly measured. Although Tax4Fun2 provides useful estimates of potential microbial functions, these predictions rely on reference genomes and assumptions regarding phylogenetic conservation of gene content. Consequently, predicted pathways may not accurately reflect the microbial community’s functional capacity, gene expression, or metabolic activity. Furthermore, 16S-based approaches cannot capture strain-level functional variation or dynamic microbial activity. Therefore, the inferred functional pathway results should be considered exploratory and hypothesis-generating rather than definitive evidence of altered microbial function. Future studies incorporating shotgun metagenomics, metatranscriptomics, and metabolomics are needed to validate these observations and elucidate underlying biological mechanisms.

### 4.2 Future directions

The variability in identified microbial features across models highlights the complexity and individualized nature of host–microbiome interactions in IBS. Future studies should focus on identifying reproducible functional pathways and integrating multi-omics approaches, including metagenomics and host transcriptomics, to better characterize mechanistic links. Larger, longitudinal cohorts are needed to validate trajectory phenotypes and microbial predictors, as well as to examine within-person dynamics over time.

From a translational perspective, these findings support the development of stratified care models in IBS. Future research should evaluate whether tailoring interventions based on microbiome and symptom profiles improves clinical outcomes. Mechanism-informed approaches targeting specific regulatory pathways—such as behavioral interventions, neuromodulation, or pharmacologic strategies—may enhance treatment precision and effectiveness.

## 5 Conclusion

Two distinct clinical phenotypes demonstrated different trajectories of symptom and neuropsychological improvement following the intervention. Although predicted functional pathway differences were not significant after correction for multiple testing, phenotype-specific microbial taxa and functional features were identified as predictors of treatment response in BART models. These findings suggest that baseline gut microbial characteristics may serve as potential biomarkers of heterogeneous treatment response in young adults with IBS. Future studies incorporating IBS subtype characterization and mechanistic validation are needed to confirm these observations.

## Data Availability

The data analyzed in this study is subject to the following licenses/restrictions: The datasets generated and analyzed in the current study are available from the NCBI SRA (https://submit.ncbi.nlm.nih.gov/subs/sra/SUB8914789/). Deidentified data will be available upon reasonable request. Requests to access these datasets should be directed to Xiaomei Cong, xiaomei.cong@yale.edu.

## Supplementary material

Supplementary Figure 1 The top 10 genera of baseline gut microbiota predict intervention response, defined as changes in pain severity, pain interference, and quality of life at 6 and 12 weeks in Phenotype A.

Supplementary Figure 2 The top 10 genera of baseline gut microbiota predict intervention response, defined as changes in pain severity, pain interference, and quality of life at 6 and 12 weeks in Phenotype B.

Supplementary Figure 3 The top 10 metabolic pathways of baseline gut microbiota that predict intervention response, defined as changes in pain severity, pain interference, and quality of life at 6 and 12 weeks in Phenotype A.

Supplementary Figure 4 The top 10 metabolic pathways of baseline gut microbiota predict intervention response, defined as changes in pain severity, pain interference, and quality of life at 6 and 12 weeks in Phenotype B.
