# Supplementary material for: Gut microbiota signatures differentiate trajectory-defined response phenotypes and predict self-management outcomes in irritable bowel syndrome
Source: Front Microbiomes. 2026 Jul 29;5:1884540. doi: 10.3389/frmbi.2026.1884540 (PMC13466233; doi:10.3389/frmbi.2026.1884540)
Supplement: Supplementary file 1 [file SupplementaryFile1.docx]

Supplementary Figure 1. The top 10 genera of baseline gut microbiota predict intervention response, defined as changes in pain severity, pain interference, and quality of life at 6 and 12 weeks in Phenotype A.

Supplementary Figure 2. The top 10 genera of baseline gut microbiota predict intervention response, defined as changes in pain severity, pain interference, and quality of life at 6 and 12 weeks in Phenotype B.

Supplementary Figure 3. The top 10 metabolic pathways of baseline gut microbiota that predict intervention response, defined as changes in pain severity, pain interference, and quality of life at 6 and 12 weeks in Phenotype A.

Supplementary Figure 4. The top 10 metabolic pathways of baseline gut microbiota predict intervention response, defined as changes in pain severity, pain interference, and quality of life at 6 and 12 weeks in Phenotype B.
